# Clinical efficacy of robot-assisted subxiphoid versus lateral thoracic approach in the treatment of anterior mediastinal tumors

**DOI:** 10.1186/s12957-023-02966-2

**Published:** 2023-03-13

**Authors:** Ziqiang Hong, Yannan Sheng, Xiangdou Bai, Baiqiang Cui, Yingjie Lu, Xusheng Wu, Tao Cheng, Dacheng Jin, Yunjiu Gou

**Affiliations:** 1 The First Clinical Medical College of Gansu University of Chinese Medicine, Gansu Provincial Hospital, Lanzhou, China; 2 Department of Thoracic Surgery, Gansu Provincial Hospital, Lanzhou, China

**Keywords:** Anterior mediastinal tumor, Laryngeal mask anesthesia, Lateral thoracic approach, Robot-assisted thoracic surgery, Subxiphoid approach

## Abstract

**Background:**

The purpose of this study was to compare the perioperative efficacy and safety of da Vinci robot-assisted thoracoscopic surgery (RATS) for treating anterior mediastinal tumors through the subxiphoid and lateral thoracic approaches under the anesthesia of nontracheal intubation (i.e., laryngeal mask airway).

**Methods:**

We retrospectively analyzed the clinical data of 116 patients with anterior mediastinal tumors treated by RATS under laryngeal mask anesthesia completed by the same operator in the Department of Thoracic Surgery, Gansu Provincial People’s Hospital, from October 2016 to October 2022. There were a total of 52 patients including 24 males and 28 females, with an average age of 45.40±4.94 years, in the subxiphoid approach (subxiphoid group). On the other hand, there were a total of 64 patients including 34 males and 30 females, with a mean age of 46.86±5.46 years in the lateral thoracic approach (lateral thoracic group). Furthermore, we have detailedly compared and analyzed the operating time, intraoperative bleeding, and total postoperative drainage in the two groups.

**Results:**

All patients in both groups successfully completed resection of the anterior mediastinal tumor without occurring perioperative death. Compared with the lateral thoracic group, the subxiphoid group has more advantages in terms of total postoperative drainage (*P*=0.035), postoperative drainage time (*P*=0.015), postoperative hospital stay (*P*=0.030), and visual analog scale (VAS) pain on postoperative days 2 (*P*=0.006) and 3 (*P*=0.002). However, the lateral thoracic group has more advantages in the aspect of docking time (*P*=0.020). There was no statistically significant difference between the two groups in terms of operative time (*P*=0.517), total operative time (*P*=0.187), postoperative day 1 VAS pain score (*P*=0.084), and postoperative complications (*P*=0.715).

**Conclusion:**

The subxiphoid approach of RATS under laryngeal mask anesthesia is safe and feasible for resecting anterior mediastinal tumors. Compared with the lateral thoracic approach, the subxiphoid approach has advantages in terms of rapid postoperative recovery and postoperative patient pain, and patient acceptance is also higher and thus is worth promoting in hospitals where it is available.

## Introduction

Early resection of anterior mediastinal tumors under RATS is often performed using a lateral thoracic approach, which can easily cause damage to the patient’s intercostal nerves and muscles, resulting in acute and chronic postoperative pain, which is not conducive to postoperative recovery [1]. In contrast, the subxiphoid approach ensures the integrity of the thorax and does not operate through the intercostal space, avoiding postoperative pain caused by injury to the intercostal nerves, easier identification of the bilateral phrenic nerves and the superior pole of the thymus, less damage to pulmonary function, and a more aesthetic surgical incision, so it has been widely used in clinical practice in recent years [2, 3]. As the technique of RATS mediastinal mass resection in our center gradually matured, we decided to continue to improve and optimize the surgical procedure on this basis and adopt non-tracheal intubation that is under laryngeal mask anesthesia to simplify the anesthesia process, shorten the operation time and recovery time, and improve the quality of life for patients after surgery. There was no available report on comparing the efficacy and safety of the subxiphoid approach versus the lateral thoracic approach in the resection of anterior mediastinal tumors by RATS under laryngeal mask anesthesia. Therefore, the aim of this study was to investigate the perioperative efficacy and safety of the subxiphoid approach versus the lateral thoracic approach in RATS under laryngeal mask anesthesia for treating anterior mediastinal tumors.

## Material and methods

### Clinical information

This is a single-center retrospective cohort study, and we included and analyzed 116 consecutive patients with anterior mediastinal tumors treated with RATS under laryngeal mask anesthesia completed by the same operator in the Department of Thoracic Surgery, Gansu Provincial People’s Hospital, from October 2016 to October 2022.

The inclusion criteria are as follows: (1) preoperative examination using enhanced computed tomography (CT) that clearly identified anterior mediastinal tumors; (2) for patients with thymoma combined with severe myasthenia gravis with stable symptom control after active medical treatment, etc. [4]; (3) no previous history of tuberculosis, pleurisy, or surgery and no preoperative relevant examination suggesting pleural thickening or adhesions; and (4) the patient’s surgical and perioperative mission and care were performed by the same medical team in our department.

Exclusion criteria are as follows: (1) middle and posterior mediastinal tumors and (2) patients with poor cardiopulmonary function or severe cardiac arrhythmias who could not tolerate surgery.

This study has been reviewed by the Ethics Committee of Gansu Provincial People’s Hospital, approval number: 2022-436. All patients signed the informed consent form for surgery before surgery.

### Surgery method (subxiphoid group)

Under general anesthesia with laryngeal mask ventilation, the patient was placed in a flat position with arms abducted, and the skin of the surgical field was routinely disinfected and a sterile surgical towel was laid.

### Perforation location

**Fig. 1:**
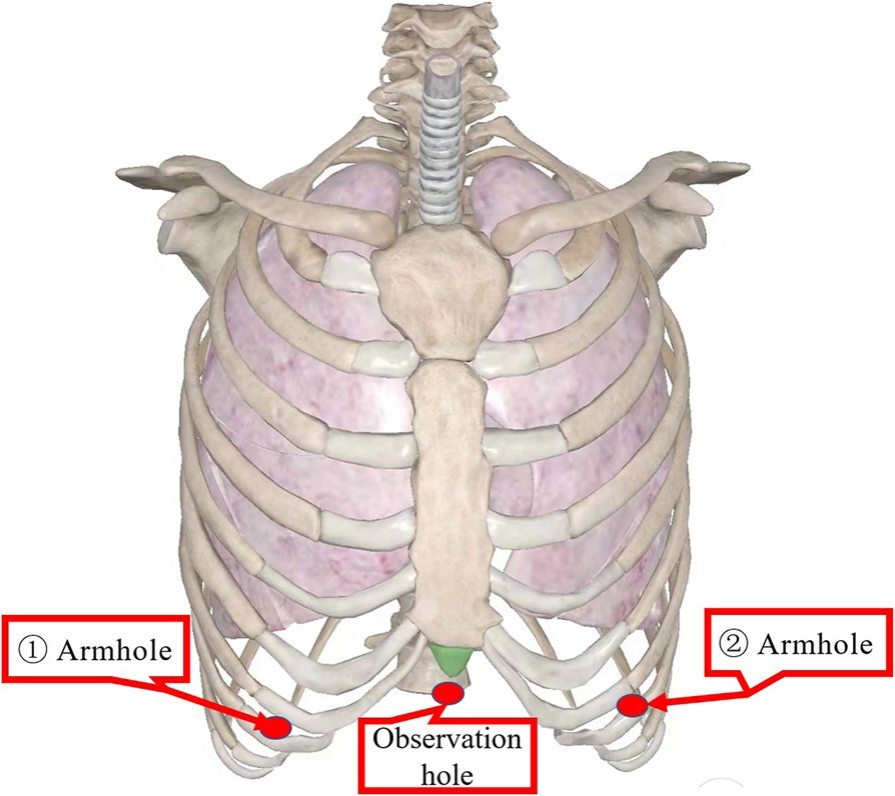
Hole layout of the subxiphoid approach

**Fig. 2:**
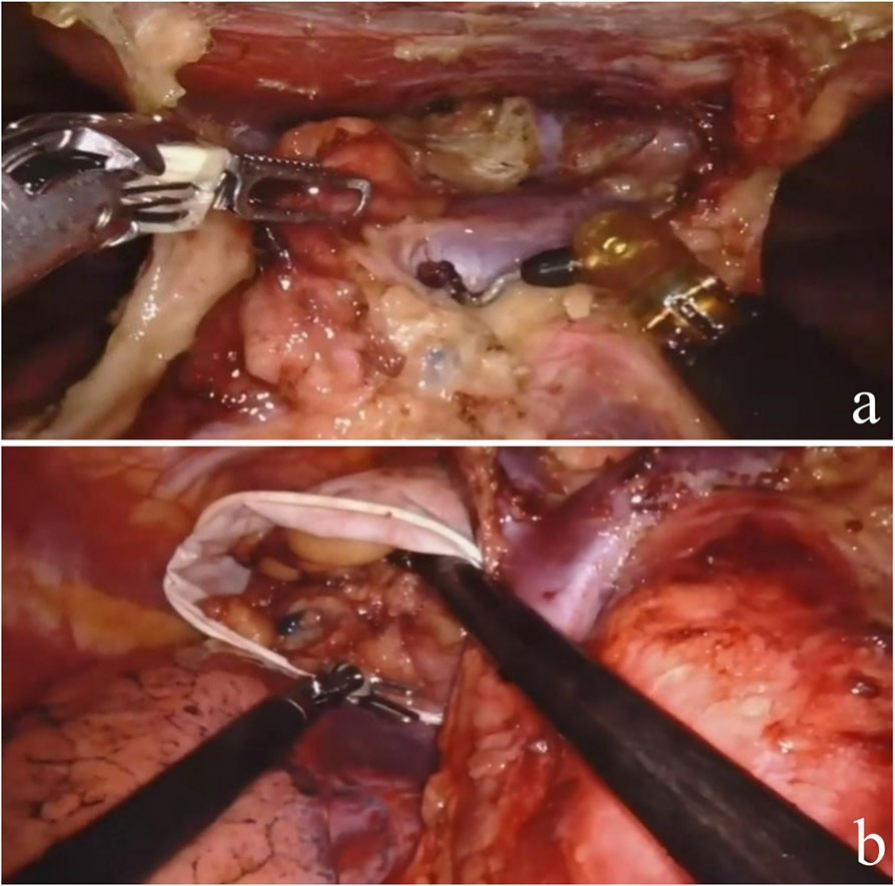
Intraoperative dissection and resection of the tumor (subxiphoid approach)

The subxiphoid incision of about 3 cm in length is the observation hole, while the robotic arm hole is set at the midclavicular line and the lower edge of the ribs on the left and right sides, respectively, and the 6th intercostal space in the midaxillary line on the one side is selected as the auxiliary operation hole if necessary (Fig. 1). CO_2_ at a pressure of 6–8 mm Hg (1 mm Hg=0.133 kPa) was introduced to establish an artificial pneumothorax to increase the posterior sternal space and fully reveal the tissue structures in the mediastinum. After bluntly separating part of the posterior sternal space, a robotic arm was placed and the chest was explored into the chest, opening the mediastinal pleura bilaterally to provide a better view and more space, and intraoperative pictures are shown in Fig. 2. In case of severe myasthenia gravis, all adipose tissues before the phrenic nerve should be removed to reduce the symptoms of myasthenia gravis and improve the patient’s prognosis. After completing the resection of the tumor, it is placed in the specimen tape and removed through the subserous incision, and the incision can be extended appropriately when the mass is large. A mediastinal drainage tube was placed through the subxiphoid incision, and the chest was closed after determining that both lungs were fully reopened, and the drainage tube was connected to negative pressure to further drain the residual air from the chest cavity, and the operation was completed.

### Surgery method (lateral thorax group)

**Fig. 3:**
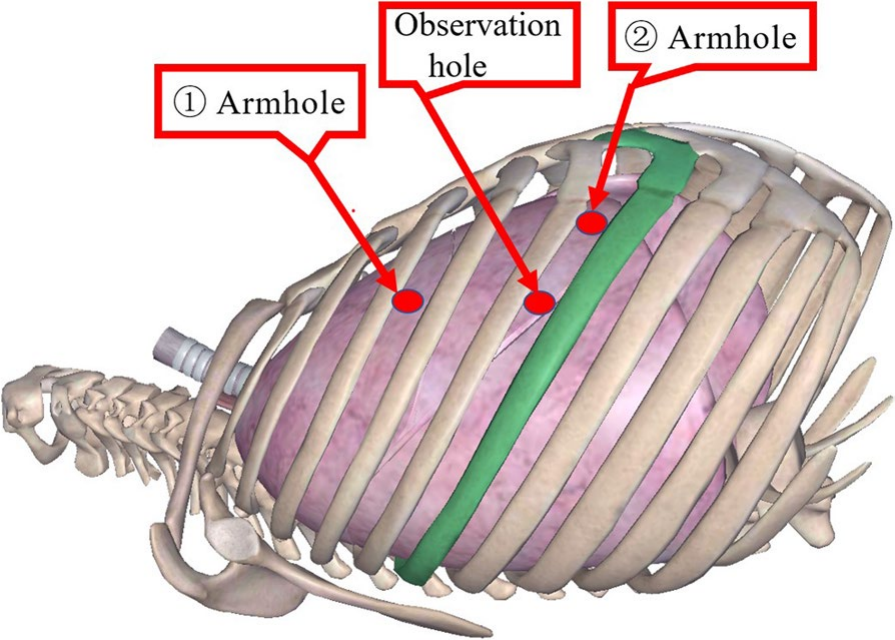
Hole layout of lateral thoracic approach

**Fig. 4:**
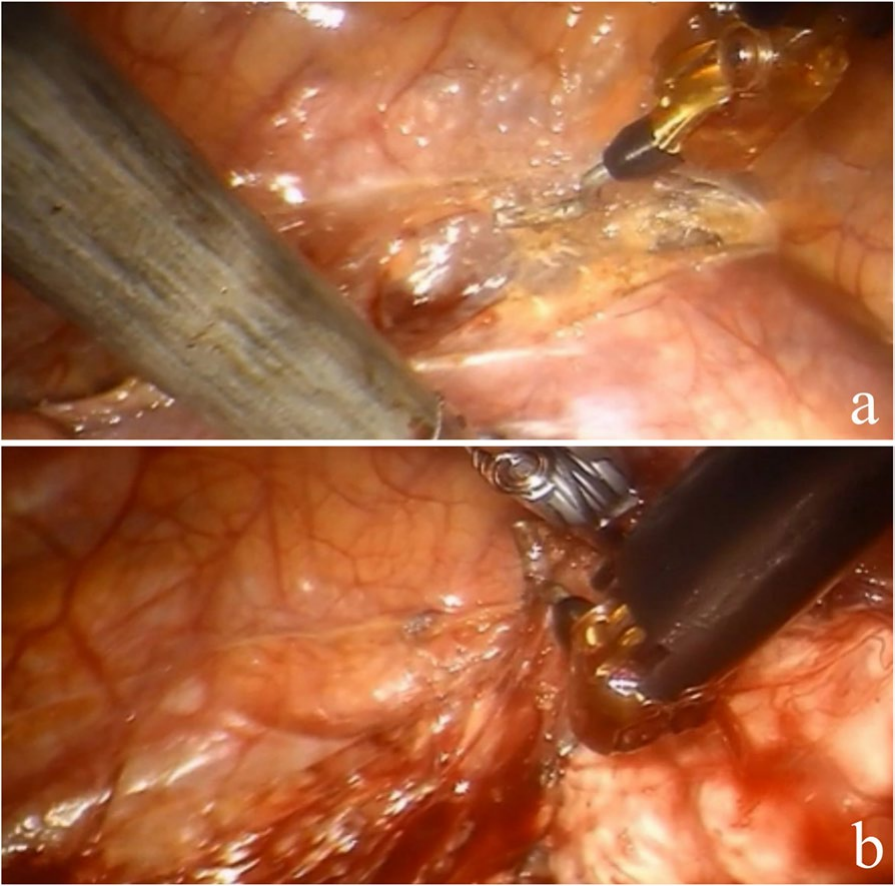
Intraoperative dissection and resection of the tumor (lateral thoracic approach)

Anesthesia and artificial pneumothorax are the same as those of the subxiphoid group. Position: generally, the upper body of the operation side can be elevated 30 to 45° in a semi-supine position (the upper limb of the affected side is abducted to expose the axilla and fixed on the anesthesia frame). The hole is set in the “5-3-5” method (Fig. 3), with “5” being the observation hole in the fifth intercostal space of the anterior axillary line on the affected side, “3” being the operation hole of arm 1 in the third intercostal space of the anterior axillary line, and “5” being the operation hole of arm 2 in the fifth intercostal space of the midclavicular line. Intraoperative pictures are displayed in Fig. 4.

### Observation indicators

Sex, age, body mass index (BMI), smoking history, comorbidities (including coronary artery disease, hypertension, and diabetes), tumor size, tumor type, and the patient’s chief complaint at the consultation. Surgical and postoperative data included the operative time, docking time, intraoperative bleeding, total postoperative drainage, postoperative lead time, postoperative hospital stay, postoperative VAS score (postoperative days 1, 2, and 3), and postoperative complications.

### Statistical analysis

SPSS 26.0 software was used for statistical analysis. If the measurement data conform to the normal distribution, it is expressed by mean ± standard deviation ($\overline{\textrm{x}}$±s), and the inter-group comparison is expressed by *t* test. If the measurement data did not conform to the normal distribution, it is expressed by the median [M(P_25_, P_75_)], and the Mann-Whitney *U* test was used for comparison between groups. Categorical variables were expressed as frequencies and percentages (%), and chi-square test or Fisher’s exact test was used for comparison between groups. Herein, *P*<0.05 was considered to be a statistically significant difference.

## Results

### Patient general information

All patients enrolled in both groups completed the surgery successfully without perioperative deaths. There was no postoperative agitation and the laryngeal mask was safely returned to the ward after removal. There was no statistically significant difference between the two groups in terms of baseline indicators such as sex, age, BMI, tumor size, and tumor type (*P*>0.05), as listed in Table 1.

**Table 1:** Comparison of clinical pathology between the two groups [cases (%)/$\overline{\textrm{x}}$±*s*]

Characteristic	Subxiphoid group (***n***=52)	Lateral thoracic group (***n***=64)	***χ***^**2**^/***t*** value	***P*** value
Sex			0.558	0.455
Male	24 (46.2)	34 (53.1)		
Female	28 (53.8)	30 (46.9)		
Age (years)	45.40±4.94	46.86±5.46	–1.490	0.139
BMI (kg/m^2^)	23.31±2.28	24.14±2.50	–1.856	0.066
Smoking history	12 (23.1)	17 (26.6)	0.186	0.666
Comorbidities				
CHD	3 (5.8)	4 (6.3)	0.012	0.914
Hypertension	7 (13.5)	10 (15.6)	0.107	0.743
Diabetes	4 (7.7)	6 (9.4)	0.103	0.748
Tumor size (cm)	3.73±0.56	3.95±0.65	–1.901	0.060
Tumor type			0.555	0.968
Benign cyst	23 (44.2)	30 (46.9)		
Thymoma	21 (40.4)	22 (34.4)		
Thymic carcinoma	2 (3.8)	3 (4.7)		
Teratoma	3 (5.8)	5 (7.8)		
Thymic hyperplasia	3 (5.8)	4 (6.2)		
General complaints			0.218	0.897
Myasthenia	7 (13.5)	7 (10.9)		
Chest tightness, chest pain, etc.	11 (21.2)	15 (23.5)		
Physical findings	34 (65.3)	42 (65.6)		

*Abbreviations*: *CHD* Coronary heart disease, *BMI* Body mass index

### Surgery results

All patients in both groups successfully completed resecting the anterior mediastinal tumor without the occurrence of perioperative death. The surgical data of the patients are given in Table 2. Compared with the lateral thoracic group, the subxiphoid group has more advantages in terms of total postoperative drainage (*P*=0.035), postoperative drainage time (*P*=0.015), postoperative hospital stay (*P*=0.030), and VAS pain on postoperative days 2 (*P*=0.006) and 3 (*P*=0.002). In the aspect of docking time, the lateral thoracic group has more advantages and the difference was statistically significant (*P*=0.020). There was no statistically significant difference between the two groups in terms of operative time (*P*=0.517), total operative time (*P*=0.187), postoperative day 1 VAS pain score (*P*=0.084), and postoperative complications (*P*=0.715).

**Table 2:** Comparison of intraoperative and postoperative indexes between the two groups [cases (%)/$\overline{\textrm{x}}$±*s*]

Characteristic	Subxiphoid group (***n***=52)	Lateral thoracic group (***n***=64)	***χ***^**2**^/***t***/***Z*** value	***P*** value
Operating time (min)	109.90±18.88	107.81±15.76	0.650	0.517
Docking time (min)	13.0 (9.0, 16.0)	10.0 (8.0, 13.0)	2.267	0.020
Total operation time (min)	122.31±18.86	118.05±15.69	1.328	0.187
Intraoperative bleeding volume (ml)	35.0 (20.0, 60.0)	40.0 (20.0, 75.0)	1.930	0.058
Total postoperative drainage (ml)	225.0 (140.0, 280.0)	240.0 (180.0, 310)	2.093	0.035
Postoperative drainage time (d)	2.0 (2.0, 3.0)	3.0 (2.0, 3.0)	2.426	0.015
Postoperative hospital stay (d)	3.5 (3.0, 4.0)	4.0 (3.0, 5.0)	2.169	0.030
Postoperative VAS pain score				
Day 1 after surgery	2.5 (2.0, 3.0)	2.8 (2.2, 3.2)	1.705	0.084
Day 2 after surgery	2.1 (1.8, 2.5)	2.5 (2.0, 3.0)	2.531	0.006
Day 3 after surgery	1.1 (1.0, 1.4)	1.7 (1.2, 2.2)	2.756	0.002
Postoperative complications	4 (8.6)	5 (21.0)	2.112	0.715
Arrhythmia	2 (3.8)	3 (4.7)		
Pleural effusion	0	1 (1.6)		
Pulmonary infection	1 (3.8)	0		
Pulmonary atelectasis	1 (1.9)	1 (1.6)		

*Abbreviation*: *VAS* Visual analog scale

## Discussion

Our center has been using the da Vinci robotic surgical system since January 2016 and routinely performed the RATS lateral thoracic approach under laryngeal mask anesthesia to treat patients with anterior mediastinal tumors in October 2016 after the RATS technique became mature. In addition, based on our experience with the subxiphoid approach in video-assisted thoracic surgery (VATS), we have also actively carried out the subxiphoid approach in RATS under laryngeal mask anesthesia to treat patients with anterior mediastinal tumors.

Previous studies have shown that both subxiphoid and lateral thoracic approaches have a high safety profile in VATS resection of anterior mediastinal tumors [5–7]. However, there are few studies comparing the efficacy and safety of the subxiphoid approach and lateral thoracic approach in RATS. However, no related study on comparing the efficacy and safety of the subxiphoid approach and the lateral thoracic approach to RATS under mask anesthesia. Therefore, in the present study, we retrospectively analyzed the short-term efficacy and safety of anterior mediastinal tumor resection by RATS under laryngeal mask anesthesia with different approaches in our single medical group.

Unlike tracheal intubation, the i-gel laryngeal mask does not enter the voice box and the following trachea, which is easy to operate, low irritation and low stress reaction, and less airway and pharyngeal complications. In this study, only one case of mild sore throat occurred in the lateral thoracic group after surgery, and no hoarseness or choking cough with drinking water occurred. It has also been shown that i-gel mask placement produces less cortisol, interleukin-6, tumor necrosis factor-α, and endocannabinoids than tracheal intubation; reduces systemic inflammation and oxidative responses [8]; and has a less hemodynamic impact [9]. However, the airway seal of the i-gel laryngeal mask is poorer than that of the tracheal intubation and cannot achieve one-lung ventilation. To ensure a good surgical view, we often add a 6–8 mm Hg artificial pneumothorax at the same time, leaving the lungs in a semi-atrophied state. The anesthesiologist does not need to deliberately small tidal volume high-frequency ventilation, if the intraoperative lung tissue atrophy on the operative side is poorly affected by the surgical field, the tidal volume can be appropriately reduced under the condition of ensuring good oxygenation, and if necessary, a bronchial occluder can be used in combination with the application of protective ventilation mode. This ensures the safety of the procedure while improving patient comfort and reducing pharyngeal and airway complications.

The results of this study showed that there was no statistically significant difference between the subxiphoid group and the lateral thoracic group in terms of total operative time, but the docking time was slightly longer in the subxiphoid group than that in the lateral thoracic group. In terms of intraoperative bleeding, there was no significant difference between the two groups. According to the author’s profound experience, the RATS lateral thoracic approach and subxiphoid approach have significant advantages in reducing intraoperative bleeding than the VATS lateral thoracic approach and subxiphoid approach we used in the past. The main reasons for the advantages of RATS in terms of surgical bleeding may be: firstly, RATS provides tenfold magnification of the three-dimensional visualized images and superior imaging quality, which facilitates intraoperative identification of various structures; secondly, RATS allows free joint movements of the robotic arm and provides seven degrees of freedom of movement through its wrist, allowing the surgeon to operate in a stable and comfortable environment, resulting in more precise dissections and avoiding nerve and artery damage; thirdly, RATS offers advantages in suturing and facilitates the operator’s management of intraoperative bleeding.

The subxiphoid approach maintains the integrity and stability of the thorax, the absence of bony structures around the xiphoid process facilitates the removal of the specimen, and it does not operate through the intercostal space, avoiding injury to the intercostal nerves and reducing postoperative pain. The results of this study showed that the subxiphoid group has more advantages in terms of total postoperative drainage, postoperative drainage time, and postoperative hospital stay compared to the lateral thoracic group. The subxiphoid approach reduces pleural injury and leakage from small lymphatic vessel breaks in the chest wall, and the pathway for pleural effusion production is reduced, which can reduce the total postoperative drainage [2]. In addition, the author believes that since patients with the subxiphoid approach have less postoperative pain, which facilitates active coughing and coughing and early bed activity, and there is less drainage and patients can remove their drains earlier. At the same time, due to earlier detubation, the patient’s subjective pain is significantly reduced, so that he or she does not experience reduced breathing due to pain and poor cooperation with postoperative mechanically assisted sputum evacuation, which in turn affects postoperative pulmonary resuscitation; since there is no restriction of bedside drainage bottles, it is easier for patients to get out of bed and reduce the incidence of crushing pneumonia due to prolonged bed rest; meanwhile, the incidence of infection is reduced because the drainage tube is connected to the outside world and bacteria have the opportunity to travel retrograde through the drainage tube and cause infection in the chest cavity [10]. In clinical practice, we have found that most patients who choose the subxiphoid approach have a faster postoperative recovery and a shorter postoperative hospital stay, which conforms to the principle of rehabilitation surgery [11].

In terms of postoperative pain VAS scores, the subxiphoid group was superior to the lateral thoracic group on postoperative days 2 and 3, while no significant difference was detected between the two groups on postoperative day 1, which is also consistent with our findings in clinical practice. There are two primary sources of postoperative pain for patients: first, pathological pain in the muscles, fascia, and nerves of the incision in the operative area, and second, postoperative pain caused by friction and pulling of the drainage tube opening. In our center, analgesia is routinely administered intravenously by an analgesic pump in the postoperative period. The prolongation of tube duration in the lateral thoracic group will aggravate the continuous irritation of the pleura and the tube opening by the drainage tube, aggravate the patient’s pain, and affect the postoperative cough and sputum, night sleep, and postoperative bed activity, thus prolonging the length of postoperative hospitalization and increasing the patient’s financial burden. In addition, the author believes that because the operating hole of the lateral thoracic group is located between the ribs in the anterior axillary line and the midclavicular line, where the intercostal muscles are thicker and the distribution of intercostal nerves and blood vessels is richer than that of the subclavian approach, the subjective pain is more significant compared with the subclavian approach. In terms of postoperative complications, the incidence of the subxiphoid group was slightly lower than that of the lateral thoracic group, but the difference between the two groups was not statistically significant.

The lateral thoracic approach in our center generally uses the right intercostal approach, which provides a relatively larger operating space on the right side of the chest compared to the left intercostal approach, a better surgical field of view, and importantly, a higher level of safety of avoiding structures such as the heart and the aortic arch [12]. If the thymic tumor is clearly protruded from the left side of the chest, the left intercostal approach is the more appropriate and safe choice [13]. In our experience, the advantages of the sword bursting down the entry path in RATS are as follows: good visual field, good exposure; more flexible robotic arm, less tremor, and safer; more complete tumor resection, more thorough clearance of anterior mediastinal fat (for thymoma, the subxiphoid approach allows observation of the thymus bilaterally, enabling more thorough and safe dissection of the thymus and its surrounding fatty tissue); easier removal of the specimen; and more aesthetic incision, less pain, and more acceptable to younger patients. In addition, although none of the cases included in this study has an intraoperative intermediate chest opening, the subxiphoid approach does not require lateral positioning and would be faster than the lateral chest approach if an emergency chest opening is required to stop bleeding in the event of unexpected hemorrhage during surgery. We have applied sternal pulling hooks in some cases, and the sternal pulling hooks greatly took advantage of the subxiphoid approach and further expanded the visual field space. In the case of obese patients, the subxiphoid approach can be limited. It has been reported that the learning curve for the subxiphoid approach in RATS for resection of anterior mediastinal tumors is 10–20 cases to reach the plateau [14]. According to our experience, the robot-assisted subxiphoid approach is more suitable for operators with experience in traditional thoracoscopic subxiphoid surgery, the learning curve of the robot-assisted subxiphoid approach is about 15 operations for those who have experience in traditional thoracoscopic subxiphoid surgery.

The present study has certain limitations and shortcomings: (1) the results may be biased due to the single-center and retrospective data source of the included studies; (2) the size of this study is small, the patients included in this study are all completed by a single medical group (same primary surgeon); (3) in this study, considering the small surgical area, the three-hole technique can better avoid mutual collision and interference between surgical instruments and is more conducive to surgical operations, and the less traumatic single-hole technique will be tried in the future; and (4) long-term survival analysis is lacking in this study, and further data refinement through follow-up is proposed.

## Conclusion

In summary, the RATS subxiphoid approach under laryngeal mask anesthesia is safe and feasible in resecting anterior mediastinal tumors. Compared with the lateral thoracic approach, the subxiphoid approach has advantages in terms of rapid postoperative recovery and postoperative patient pain, and patient acceptance is also higher, which is worth promoting in hospitals with available conditions.

## Data Availability

The datasets used and/or analyzed during the current study are available from the corresponding author on reasonable request.

## Abbreviations

RATS: Robot-assisted thoracoscopic surgery
VAS: Visual analog scale
CT: Computed tomography
BMI: Body mass index
CHD: Coronary heart disease
VATS: Video-assisted thoracic surgery
